# Evolutionary history of Mexican domesticated and wild *Meleagris gallopavo*

**DOI:** 10.1186/s12711-018-0388-8

**Published:** 2018-04-17

**Authors:** Gabriela Padilla-Jacobo, Horacio Cano-Camacho, Rigoberto López-Zavala, María E. Cornejo-Pérez, María G. Zavala-Páramo

**Affiliations:** 1 Centro Multidisciplinario de Estudios en Biotecnología, FMVZ, Universidad Michoacana de San Nicolás de Hidalgo, Km. 9.5 Carretera Morelia-Zinapécuaro, Posta Veterinaria, C.P. 58000 Tarímbaro, Michoacán Mexico; 2 Facultad de Medicina Veterinaria y Zootecnia, Universidad Autónoma de Tamaulipas, Km. 5, Carretera a Cd. Mante S/S, C.P. 87276 Ciudad Victoria, Tamaulipas Mexico

## Abstract

**Background:**

The distribution of the wild turkey (*Meleagris gallopavo*) extends from Mexico to southeastern Canada and to the eastern and southern regions of the USA. Six subspecies have been described based on morphological characteristics and/or geographical variations in wild and domesticated populations. In this paper, based on DNA sequence data from the mitochondrial D-loop, we investigated the genetic diversity and structure, genealogical relationships, divergence time and demographic history of *M. gallopavo* populations including domesticated individuals.

**Results:**

Analyses of 612 wild and domesticated turkey mitochondrial D-loop sequences, including 187 that were collected for this study and 425 from databases, revealed 64 haplotypes with few mutations, some of which are shared between domesticated and wild turkeys. We found a high level of haplotype and nucleotide diversity, which suggests that the total population of this species is large and stable with an old evolutionary history. The results of genetic differentiation, haplotype network, and genealogical relationships analyses revealed three main genetic groups within the species: mexicana as a population relict (C1), merriami (C2), and mexicana/intermedia/silvestris/osceola (C3). Haplotypes detected in domesticated turkeys belong to group C3. Estimates of divergence times agree with range expansion and diversification events of the relict population of *M. gallopavo* in northwestern Mexico during the Pliocene–Pleistocene and Pleistocene–Holocene boundaries. Demographic reconstruction showed that an expansion of the population occurred 110,000 to 130,000 years ago (Kya), followed by a stable period 100 Kya and finally a decline ~ 10 Kya (Pleistocene–Holocene boundary). In Mexico, the Trans-Mexican Volcanic Belt may be responsible for the range expansion of the C3 group. Two haplotypes with different divergence times, MGMDgoB/MICH1 and MICH2, are dominant in domesticated and commercial turkeys.

**Conclusions:**

During the Pleistocene, a large and stable population of *M. gallopavo* covered a wide geographic distribution from the north to the center of America (USA and Mexico). The mexicana, merriami, and mexicana/intermedia/silvestris/osceola genetic groups originated after divergence and range expansion from northwestern Mexico during the Pliocene–Pleistocene and Pleistocene–Holocene boundaries. Old and new maternal lines of the mexicana/intermedia/silvestris/osceola genetic group were distributed within the Trans-Mexican Volcanic Belt where individuals were captured for domestication. Two haplotypes are the main founder maternal lines of domesticated turkeys.

**Electronic supplementary material:**

The online version of this article (10.1186/s12711-018-0388-8) contains supplementary material, which is available to authorized users.

## Background

*Meleagris gallopavo* is an original neartic species with a distribution that extends from Mexico to southeastern Canada and to the eastern and southern regions of the USA [1, 2]. Six subspecies of *M. gallopavo* have been described based on their geographical distribution and morphological characteristics such as size, coloration or iridescence of plumage, color of the legs, and color of the tip and base of the feathers, i.e. (1) *M. g. gallopavo* (domesticated) described by Linnaeus in 1758, (2) *M. g. silvestris* (Silvestre) described by Vieillot in 1817, (3) *M. g. mexicana* (Gould) described by Gould in 1856, (4) *M. g. intermedia* (Río Grande) described by Sennett in 1879, (5) *M. g. osceola* (Florida) described by Scott in 1890, and (6) *M. g. merriami* (Merriam) described by Nelson in 1900 (for more details see [2–5]). *Meleagris gallopavo* is the one and only important domesticated animal species of North American origin [6]. Molecular studies based on mtDNA have suggested that the domesticated turkey is representative of the extinct wild subspecies *M. g. gallopavo* [7, 8]. Knowledge from historical registries indicates that different prehispanic Mexican groups such as the Purepecha, Huicholes and other ethnic groups of domesticated turkeys were present between 200 and 700 BC [9]. Leopold [6] and Nelson [10] proposed that domestication occurred in the highlands of Michoacan, Mexico, and according to Schorger [3] domesticated turkey stocks were established by at least ca. 200 BC to 700 AD within the Tehuacan Valley (Puebla), with bones dated from approximately 700 AD being identified in Guatemala.

There are few molecular genetic studies on domesticated turkeys from rural Mexican communities. As far as we know, there is only one analysis that used microsatellite markers to analyze domesticated turkey populations from the five physiographic regions of Michoacan in Mexico and that revealed three genetically distinct groups [11]. Some studies focused on commercial or heritage turkeys, and for example, a microsatellite analysis showed that the commercial turkey is closer to the heritage Narragansett, Bourbon Red, Blue Slate turkeys than to the Spanish Black and Royal Palm turkeys [12]. A genomic study that included seven commercial lines, three samples of wild turkeys from Chihuahua in Mexico, and the heritage Beltsville Small White, Royal Palm and Narraganset varieties revealed that all commercial lines shared the same origin and that specific haplotypes may have been selected in the modern domesticated turkey [13]. Other studies have focused on the analysis of the diversity between subspecies and conservation of wild populations [5, 14, 15]. Finally, an analysis of samples from bones and coprolites from archaeological sites in the southwestern USA and from the six proposed *M. gallopavo* subspecies using mitochondrial markers proposed two sites of turkey domestication that each involve wild turkey populations i.e. (1) *M. g. gallopavo* in south-central Mexico and (2) *M. g. intermedia*/*silvestris* with a subsequent introduction of domesticated stocks in the southwestern USA [7].

For agriculturally important species such as chicken (*Gallus gallus*) [16–19], duck (*Anas platyrhynchos*) [20–23], cattle (*Bos taurus*) [24–26], and pigs [27, 28], phylogenetic and genealogical molecular analyses have helped to better understand the process of their domestication and to demonstrate the origin and inter- or intraspecific relationships of these species. Moreover, the use of mtDNA sequences in phylogeography analyses has been extensively tested and offers a highly sensitive method to analyze evolutionary processes [29]. Currently, they are the most widely used markers for such studies in vertebrates.

In this study, our aim was to investigate the genetic diversity and structure, genealogical relationships, divergence times and the demographic history of *M. gallopavo*, by putting emphasis on domesticated individuals to reconstruct the evolutionary history of this species. For these analyses, we used sequences of the mtDNA D-loop from domesticated, commercial and wild turkeys.

## Methods

### Sample collection

Blood and tissue samples were collected from domesticated and wild turkey populations between 2001 and 2011. For each individual, blood samples of 0.1 to 0.2 mL were taken from the brachial vein or tissue fragments as large as a grain of rice were obtained and placed in 2 mL vials with 0.5 mL of storage and lysis buffer (100 mM Tris pH 8.0, 100 mM EDTA pH 8.0, 10 mM NaCl and 2% SDS), saturated with salt-DMSO for tissue samples [30]. Then, samples were stored at room temperature during transport (about 1 week) and subsequently stored at 4 °C until further analysis.

**Fig. 1:**
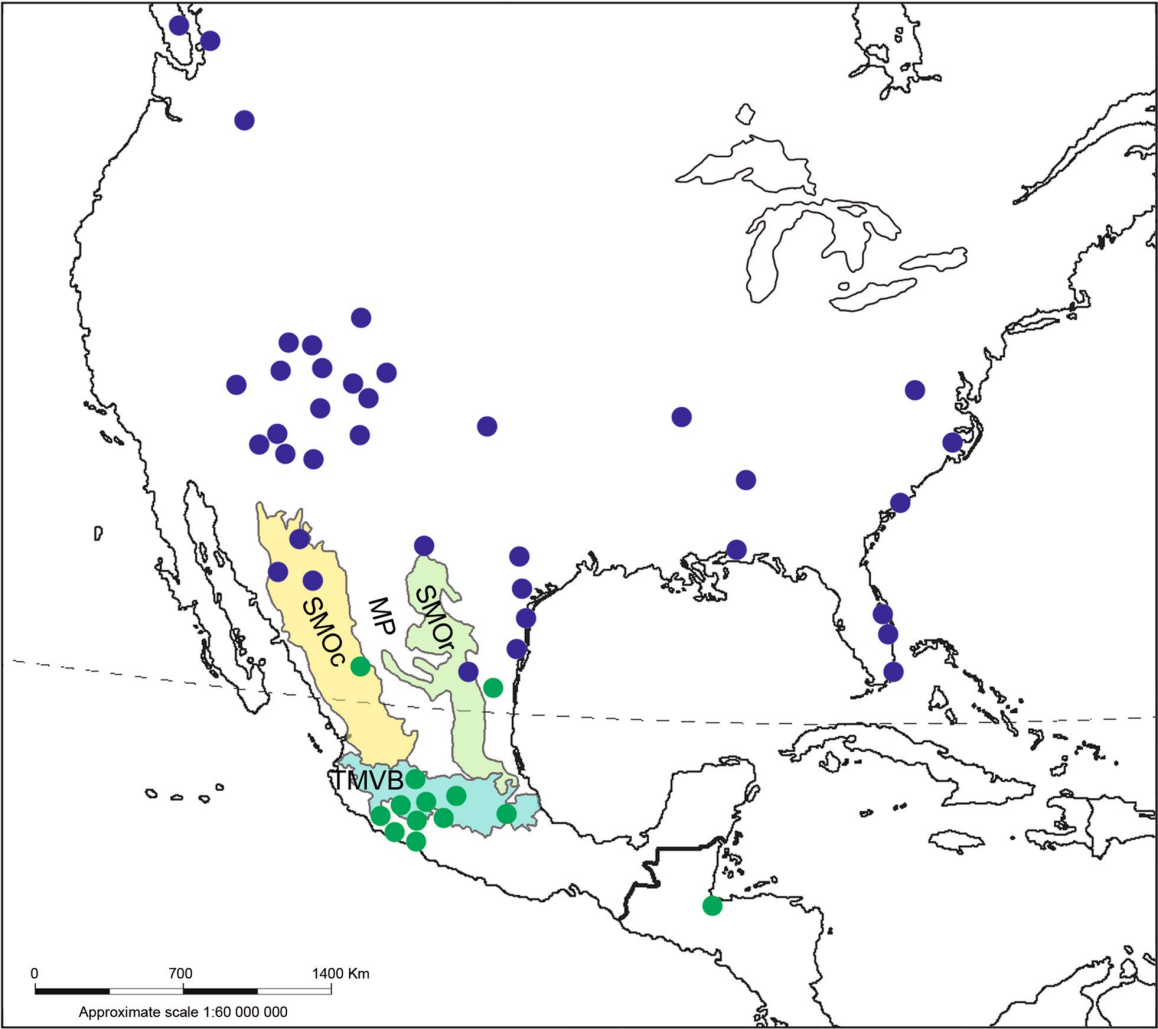
Geographical origin of the turkey samples used for this study. Localities of sampling of turkeys for this study (green dots), and locations of the NCBI GenBank database sequences (blue dots); the shaded polygons correspond to the following geographical regions of Mexico: SMOc = Sierra Madre Occidental (yellow), SMOr = Sierra Madre Oriental (green), TMVB = Trans-Mexican Volcanic Belt (blue), and the MP = Mexican Plateau (not shaded)

Samples were deposited in the Collection of Biological Samples of the Centro Multidisciplinario de Estudios en Biotecnologia (CMEB) of the Universidad Michoacana de San Nicolas de Hidalgo. A total of 187 samples were available for analysis: 161 originated from domesticated populations of *M. gallopavo* collected in Mexican rural communities from different localities in Puebla and from the five physiographic regions in Michoacan (Bajio, Trans-Mexican Volcanic Belt, Balsas, Sierra, and Costa) [31] (see Fig. 1 and Table 1); four samples of domesticated turkeys were obtained from the Izabal department in the northeastern region of Guatemala; nine samples of the commercial line Bronze were collected on a farm in Ario de Rosales, Michoacan, Mexico (Fig. 1, Table 1); nine samples of *M. g. mexicana* individuals were obtained from a management unit for wildlife conservation in Canatlan, Durango in Mexico, a population that was reintroduced from Yecora, Sonora in Mexico; and four samples of *M. g. intermedia* were donated by hunters that held a permission to hunt in Villa de Casas, Tamaulipas in Mexico (Fig. 1, Table 1).

**Table 1:** Localities where samples of wild and domesticated turkeys from Mexico and Guatemala were collected

Country	Sample types	State	Locality	Sample size
Mexico	*M. g. mexicana*	Durango	Canatlan (UMA El Durangueño)	9
	*M. g. intermedia*	Tamaulipas	Villa de Casas	4
	Domesticated	Michoacan	Bajio Region (Penjamillo, Indaparapeo, Zacapu, La Piedad, Jimenez, Capula, Churintzio, Zamora)	33
			Trans-Mexican Volcanic Belt Region (Zitacuaro, Cuitzeo, Benito Juarez, Tuxpan, Tacambaro, Ario de Rosales, Salvador Escalante)	31
			Balsas Region (Churumuco, Huetamo, Tiquicheo, Tepalcatepec, Tuzantla, Nuevo Urecho, Buenavista, Apatzingan)	26
			Sierra Region (Tumbiscatio, Arteaga, Aguililla)	28
			Costa Region (Coahuayana, Aquila, Arteaga)	26
		Puebla	Rafael Lara Grajales (Benito Juarez Ejido)	1
			Totimehuacan (San Baltazar Azumiatla)	5
			Soltepec (San Mateo)	7
			Hueyotlipan (Sto. Tomas Hueyotlipan)	4
	Commercial line Bronze	Michoacan	Ario de Rosales	9
Guatemala	Domesticated	Izabal	Izabal	4
Total				187

### DNA extraction and amplification

DNA was extracted from tissue and blood samples using the phenol-free method described by FitzSimmons [32]. The mtDNA D-loop sequence was amplified using the oligonucleotides NAU313 (5′ GCCACCTGTGAAGAAGCC 3′) and NAU185 (5′ ACGGCTTGAAAAGCCATTGTTGT 3′) [5]. PCR reactions were performed in a total volume of 25 µL as follows: PCR buffer 1× (20 mM Tris–HCl pH 8.4, 50 mM KCl, 1.5 mM MgCl_2_), 200 mM of each dNTP, 10 pmol of each oligonucleotide, 1.5 U Platinum *Taq* polymerase (Invitrogen) and 50 ng of DNA. The reaction mixtures were placed in a thermocycler (Gene Amp 2700, Applied Biosystems) under the following amplification conditions: 95 °C for 5 min, followed by 30 cycles of 95 °C for 1 min, 60 °C for 1 min and 72 °C for 2 min, with a final extension at 72 °C for 8 min. DNA sequencing was performed using the dideoxy technique on both strands [33] using the commercial service Macrogen USA. In addition to these 187 sequences, we analyzed 425 sequences from wild and domesticated individuals that were obtained from the NCBI GenBank database (Table 2), which amounted to 612 sequences. Figure 1 shows the location of the individuals sampled for this study and the geographical origin of the sequences obtained from the NCBI GenBank database.

**Table 2:** Nucleotide sequences of D-loop from *M. gallopavo* and codes used in the analyses

Population/subspecies	GenBank accession number	Code in this study	Population/subspecies	GenBank accession number	Code in this study
*M. g. mexicana*	AF486960	MGM60	*M. gallopavo, a*rchaeological samples	GQ303159	MGarq59
	AY037888	MGM88		GQ303160	MGarq60
*M. g. silvestris*	AF486901	MGS01		GQ303161	MGarq61
	AF486902	MGS02		GQ303163	MGarq63
	AF486905	MGS05	*M. g. gallopavo* 1903	GQ303164	MGarq64
	AF486911	MGS11		GQ303165	MGarq65
	AF486914	MGS14	*M. g. gallopavo*	EF153719	MG19
	AF486918	MGS18		AF172952	MG52
	AF486921	MGS21		AJ297180	MG80
	AF486927	MGS27	*M. g. intermedia*	AF487103	MGI03
	AF172947	MGS47		AF487117	MGI17
	AF172953	MGS53		AF487058	MGI58
	AF172954	MGS54		AF487059	MGI59
	AF172957	MGS57		AF487060	MGI60
	AF172958	MGS58		AF487062	MGI62
	AF172960	MGS60		AF487063	MGI63
	AF172961	MGS61		AF487065	MGI65
	AF486875	MGS75		AF487067	MGI67
	AF486876	MGS76		AF487071	MGI71
	AF486877	MGS77		AF487072	MGI72
	AF486885	MGS85		AF487077	MGI77
	AF486887	MGS87		AF487085	MGI85
	AF486889	MGS89		AF487094	MGI94
	AF486895	MGS95	*M. g. merriami*	AF487006	MGMer06
	AF486898	MGS98		AF487010	MGMer10
*M. g. oceola*	AF486931	MGO31		AF487023	MGMer23
	AF486938	MGO38		AF487025	MGMer25
	AF486944	MGO44		AF487039	MGMer39
	AF486949	MGO49		AF487041	MGMer41
	AF486951	MGO51		AF487042	MGMer42
	AF486953	MGO53		AF172948	MGMer48
	AF486956	MGO56		AF172964	MGMer64
	AF486959	MGO59		AF486985	MGMer85
*M. gallopavo,* archaeological samples	GQ303154	MGarq54		AF486986	MGMer86
	GQ303155	MGarq55		AF486997	MGMer97
	GQ303156	MGarq56		AF486999	MGMer99
	GQ303157	MGarq57	*M. ocellata*	AF487120	AF487120Aocellata
	GQ303158	MGarq58		AF487121	AF487121Aocellata

### Genetic diversity and differentiation

Sequence editing, alignment, and construction of data matrices were carried out with Sequencher v4.1 [34] and PhyDE [35]. The number of haplotypes (H), polymorphic sites (S), and nucleotide (π) and haplotype (Hd) diversity estimates for the domesticated and wild populations were calculated with DnaSP v5 [36]. Analysis of molecular variance (AMOVA) [37] was used to calculate genetic variation and genetic differentiation between populations by performing 10,000 permutations. In addition, computed pairwise comparisons of *F*_ST_ values with 1000 permutations were obtained with ARLEQUIN v3.1 [38].

### Genealogical relationships between haplotypes

To establish genealogical relationships between haplotypes and their frequencies, a haplotype network was constructed using the median-joining method [39] with the software NETWORK v4.6.0.0 [40] and setting default parameters. The relationships between haplotypes were also analyzed using phylogenetic inference. Matrices for these analyses included haplotypes that were identified in this study and haplotypes for each subspecies that are reported in the NCBI GenBank database (Table 2). The sister species *Meleagris ocellata* was included as outgroup (Table 2). Models of molecular evolution were evaluated with jModelTest v2.1.1 [41] and selected using the corrected Akaike Information Criterion (cAIC) [42]. The best model obtained using this criterion was Hasegawa, Kishino and Yano [43] + Invariant sites, i.e. HKY+I.

Reconstructions of genealogical relationships were generated using maximum likelihood (ML) and Bayesian inference (BI) frameworks with RAxML v8 [44] and MrBayes v3.2 [45], respectively. Branch support values were estimated by bootstrap analysis (BP) of 500 replicates and by calculating posterior probabilities (PP). MrBayes was run with the following parameters: four independent runs of four chains each (one cold chain and three hot chains) for 10 million generations, sampling one tree every 1000 generations. Trees and parameters were summarized after discarding 25% of the data as burn-in. The remaining trees were summarized as a majority consensus tree and visualized using FigTree v1.4.0 [46].

### Estimation of divergence times and rates of molecular evolution

The data matrix included two sequences of *M. ocellata* and one sequence of *Gallus gallus* (GenBank Access HQ022888.1). Divergence times were estimated using BEAST v1.7.4 [47]. An uncorrelated lognormal relaxed clock model was selected with the HKY+I model of evolution. Because of the nature of the data, a tree prior with a coalescent model assuming a constant population size was used [48]. One calibration point with a lognormal prior distribution (mean = 0.0, standard deviation (SD) = 1.0, offset = 2.6) and the oldest *M. gallopavo* fossil (2.6 Mya) that is registered in the PaleoDB fossil database (82,258) [49] were used. Markov chain Monte Carlo (MCMC) analyses were run for 10 million generations, sampling one tree every 1000 generations. The results were summarized using TreeAnnotator v1.7.4 [47]. Ten percent of the trees were discarded as burn-in, and the remaining trees were summarized as a maximum clade credibility tree including average divergence times and their associated 95% high posterior densities (HPD). Trees were visualized using FigTree v1.4.0 [46]. We used *K *= *r*/2*t* to estimate the rate of substitutions per site [50].

### Demographic history

We used a Bayesian skyline plot [51] that was estimated by BEAST v1.7.4 [47] and mismatch distribution [52] to infer demographic history. Five independent runs with 30 million generations were conducted. The substitution model HKY+I with empirical base frequencies, was used with an uncorrelated lognormal relaxed clock model and a piecewise-constant coalescent Bayesian skyline tree prior with 10 starting groups. Trees and parameters were sampled every 1000 iterations, with a burn-in of 10%. The results of each run were combined in LogCombiner v1.7.4 [47] and the result was visualized by using TRACER 1.5 [53]. In addition, mismatch distributions were obtained with the ARLEQUIN software package [38]. Mismatch distributions were calculated using the sudden expansion model [54] with 1000 parametric bootstraps. The sum of squared deviations (SSD) and Harpending´s raggedness index (Hri) were calculated to assess the validity of the sudden expansion assumption.

## Results

### Sequence analysis, genetic diversity and differentiation

From the DNA samples of *M. gallopavo* collected for this study, we obtained 187 sequences (556 to 672 bp long) of the mtDNA D-loop that were registered in GenBank (Accession numbers: MF161996 to MF162182). Fifteen haplotypes were identified within the domesticated and wild turkey individuals analyzed in this study with an overall moderate Hd and low π (Table 3), among which 11 were found in the domesticated turkeys from Mexico, Guatemala, and the commercial line Bronze with nine polymorphic sites, moderate Hd and low π. For the *M. g. mexicana* individuals, we detected five haplotypes with eight polymorphic sites, high Hd and low π. Finally, only one haplotype was identified in *M. g. intermedia*.

**Table 3:** Genetic diversity indices for the domesticated/commercial, *M. g. mexicana,* and *M. g. intermedia* turkeys included in this study

Population	n	nt	H	Hd	π	S
Domesticated/commercial	174	549	11	0.558	0.00153	9
*M. g. mexicana*	9	632	5	0.806	0.00483	8
*M. g. intermedia*	4	637	1	0	0	0
Total	187	535	15	0.586	0.00198	16

*n* number of individuals, *nt* number of characters considered in the matrix, *H* number of haplotypes, *Hd* haplotype diversity, *π* nucleotide diversity, *S* number of polymorphic sites

For the domesticated population from Mexico, we detected two dominant haplotypes designated MICH1 (n = 107) and MICH2 (n = 44) present in 61.49 and 25.28% of the individuals in the population, respectively. Interestingly, 95 domesticated individuals from Michoacan, eight from Puebla, all those from Guatemala, one from the commercial line Bronze, all *M. g. intermedia* individuals, and one *M. g. mexicana* individual, which was originally designated as carrying the MGMDgoB haplotype, shared the MICH1 haplotype. Thus, considering that MGMDgoB and MICH1 are the same haplotype or maternal line, it was hereafter designated as the MGMDgoB/MICH1 haplotype. In addition, 36 other domesticated individuals from Michoacan, two from Puebla, and six from the commercial line Bronze shared the MICH2 haplotype. These results revealed that many of the domesticated turkeys of Mexico and Guatemala and the individuals of the Bronze commercial line shared the same haplotypes; therefore, in the following analyses, they were treated as a single group called domesticated/commercial.

Next, to corroborate and strengthen our results, the mtDNA D-loop sequences of all domesticated (described as *M. gallopavo*) and wild individuals reported in the NCBI GenBank database were included in the following analyses (Table 2). The total population analyzed (n = 612) showed overall high Hd and π (Table 4). All the sequences of domesticated turkeys that were present in the NCBI GenBank database were included in the domesticated/commercial group. The analysis of domesticated/commercial turkeys showed moderate Hd and low π (Table 4). Among the wild populations, diversity levels varied with the *M. g mexicana* population showing the lowest Hd and π levels, and the *M. g. silvestris* and *M. g. merriami* populations the highest levels (Table 4).

**Table 4:** Genetic diversity indices for each population

Population	n	H	S	Hd	π	D-Tajima
Domesticated/commercial	194	13	12	0.569	0.00162	− 1.60009 (NS, 0.10 > *P* > 0.05)
*M. g. intermedia*	66	13	13	0.851	0.00530	− 0.43092 (NS, *P* > 0.10)
*M. g. merriami*	217	18	15	0.628	0.00826	1.09874 (NS, *P* > 0.10)
*M. g. mexicana*	36	6	9	0.308	0.00209	− 1.72617 (NS, *P* < 0.10)
*M. g. osceola*	29	8	8	0.813	0.00402	− 0.41858 (NS, *P* > 0.10)
*M. g. silvestris*	70	22	23	0.913	0.00485	− 1.71251 (NS, 0.10 > *P* > 0.05)
All samples	612	64	46	0.888	0.00774	− 1.34597 (NS, *P* > 0.10)

*n* number of individuals, *H* number of haplotypes, *S* polymorphic sites, *Hd* haplotype diversity, *π* nucleotide diversity

Analysis of the genetic differentiation between the domesticated/commercial group and the wild populations showed that there is differentiation among these populations (Table 5). The highest differentiation was observed between domesticated/commercial turkeys and *M. g. mexicana*. In addition, the lowest genetic differentiation values were found between *M. g. intermedia, M. g. osceola* and *M. g. silvestris*. Based on these results, we defined three groups of *M. gallopavo*: mexicana, merriami, and intermedia/silvestris/osceola/domesticated/commercial. The distribution of genetic variation obtained by AMOVA without a priori defined groups revealed that the genetic variation was highest within populations (Table 6). The percentage of genetic variation among populations and the high fixation index indicated a structure with subpopulations within species. When we divided the population into three groups, the percentage of genetic variation between them was equal to 31.14%. The fixation index *F*_CT_ reached a high value indicating a high level of genetic differentiation among groups (Table 6).

**Table 5:** Pairwise genetic differentiation (*F*_ST_) of populations

	*M. g. merriami*	Domesticated/commercial	*M. g. silvestris*	*M. g. intermedia*	*M. g. mexicana*	*M. g. osceola*
*M. g. merriami*	–					
Domesticated/commercial	0.50550	–				
*M. g. silvestris*	0.25394	0.49995	–			
*M. g. intermedia*	0.23189	0.35257	0.14670	–		
*M. g. mexicana*	0.43530	0.85891	0.65913	0.56566	–	
*M. g. osceola*	0.25278	0.54871	0.12507	0.09326	0.68575	–

**Table 6:** Summary AMOVA without a priori defined groups and in three groups

Source of variation	d.f.	Sum of squares	Variance components	Percentage of variation	Fixation index
*Without a priori defined groups*					
Among population	5	387.469	0.83993	43.98	
Within population	606	648.225	1.06968	56.02	*F*_ST_ = 0.43984
Total	611	1035.694	1.90961	100	
*In three groups* ^a^					
Among groups	2	299.146	0.65399	31.14	*F*_CT_ = 0.31139
Among populations within groups	3	88.323	0.37655	17.93	*F*_SC_ = 0.26036
Within populations	606	648.225	1.06968	50.93	*F*_ST_ = 0.49068
Total	611	1035.694	2.10021	100	

^a^In three groups: (1) mexicana, (2), merriami, and (3) intermedia/osceola/silvestris/domesticated/commercial

### Haplotype network

**Fig. 2:**
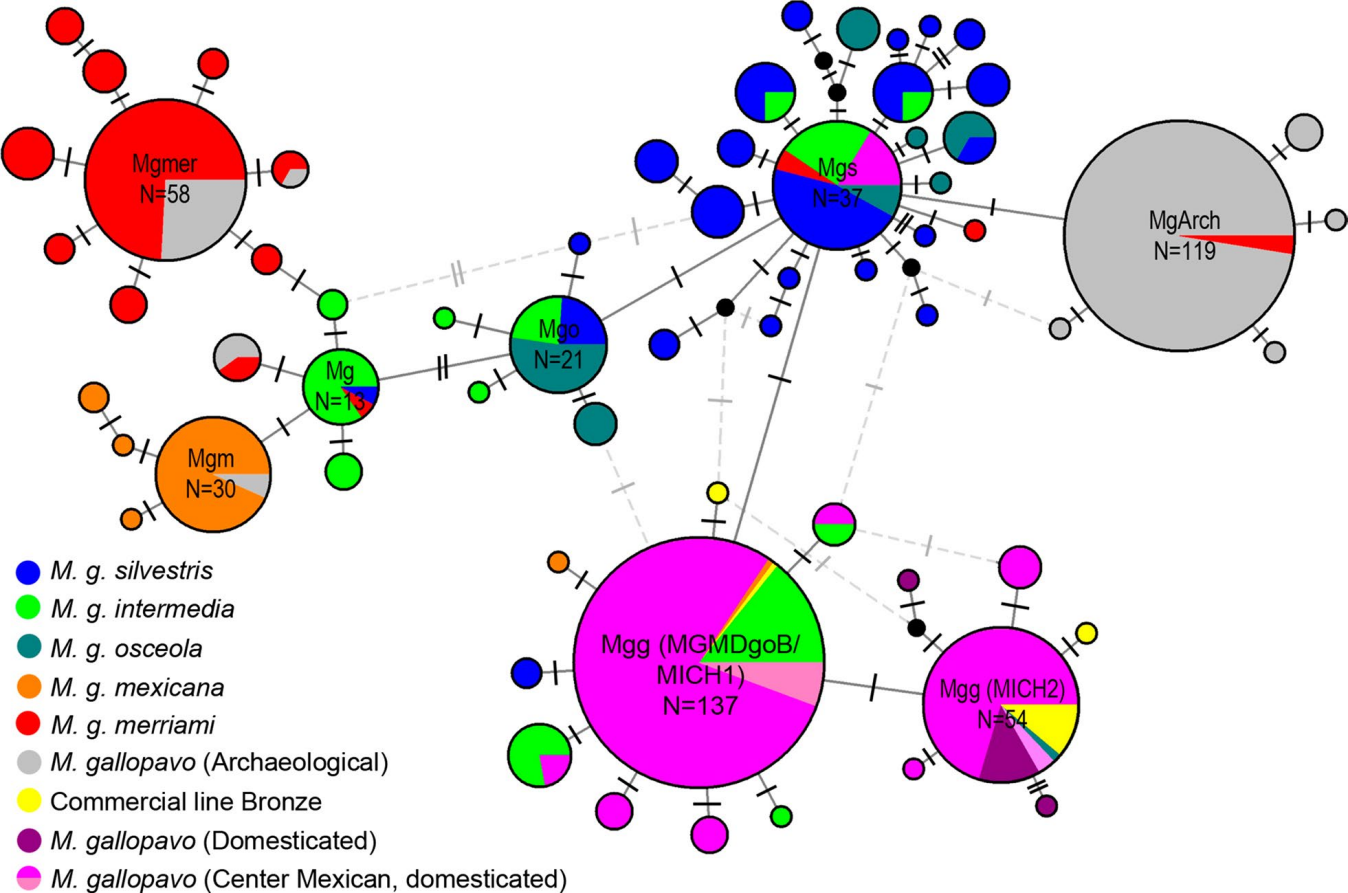
Median-joining haplotype network. The relationships between haplotypes identified in domesticated and wild *M. gallopavo*, including 612 individuals and 64 haplotypes are shown. The size of the circles is proportional to the haplotype frequency. Black circles represent hypothetical intermediates or unsampled haplotypes. Dotted gray lines indicate loops that were broken according to Crandall and Templeton [70]

We constructed a haplotype network to visualize the relationships between haplotypes and their frequencies for the 612 domesticated/commercial and wild turkey sequences (see Additional file 1: Table S1). The analyses revealed 64 haplotypes that differed from each other by a small number of mutations. The network (Fig. 2) shows eight haplogroups, each with a dominant haplotype. Four mexicana haplotypes clustered with those obtained from the NCBI GenBank database, forming a haplogroup with 34 individuals that shared the dominant haplotype Mgm (Fig. 2). A haplogroup that derives from haplotype Mgm has the dominant haplotype Mg, which is shared by intermedia, merrami, and silvestris samples. Merriami turkeys integrate a haplogroup with the dominant haplotype Mgmer that is shared with the archeological samples [7]. Haplotypes of the intermedia individuals are dispersed and shared with domesticated/commercial, oceola, and silvestris turkeys. A haplogroup that contains mainly silvestris turkeys showed a dominant haplotype (Mgs) that is shared with domesticated (from Puebla, Mexico), osceola, intermedia, and merriami individuals, and with peripheral haplotypes forming a star. Moreover, Mgs is related to the dominant haplotypes Mgo, MGMDgoB/MICH1, and MgArch through one mutation. Two haplogroups were linked with the dominant haplotypes MGMDgoB/MICH1 and MICH2. Haplotype MGMDgoB/MICH1 is shared by domesticated turkeys from Mexico, Guatemala, Canada, and the USA, individuals of the commercial line Bronze and wild turkeys in the intermedia and mexicana populations. It was also detected in individuals that were previously identified as wild *M. g. gallopavo,* for which samples were collected in 1903 in Veracruz and Michoacan, Mexico [7]. Additional haplotypes derived from MGMDgoB/MICH1 are present in domesticated turkeys of Michoacan, individuals of the commercial line Bronze, and mexicana, intermedia, and silvestris turkeys. Haplotype MICH2 is shared between domesticated turkeys from Mexico and Canada, most individuals of the commercial line Bronze, an osceola individual, and it corresponds to the same haplotype of an individual that was identified as wild *M. g. gallopavo* from Michoacan and collected in 1903 [7]. Derived from the MICH2 haplotype, peripheral haplotypes were identified that are present in the commercial line Bronze and domesticated turkeys from Michoacan (Fig. 2).

### Genealogical relationships

**Fig. 3:**
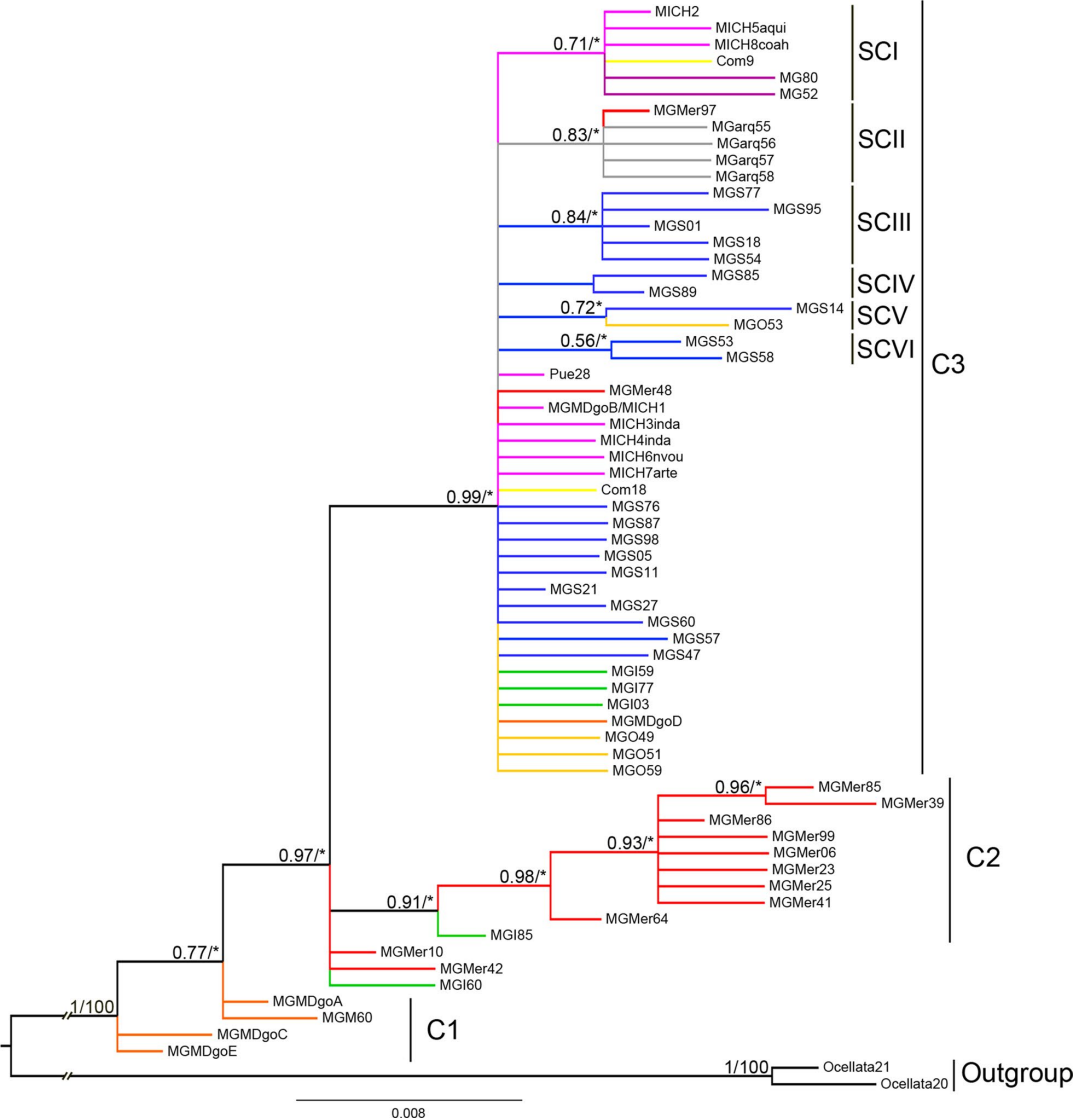
Genealogy of *M. gallopavo* obtained with Bayesian inference (BI) and maximum likelihood (ML) analyses. Estimates were based on 441 characters of *M. gallopavo* mitochondrial control region DNA sequences. The out-group is the sister species *M. ocellata*. Names of distinct clades/groups are indicated. Values over the branches represent posterior probabilities and bootstrap values (PP/BP). (*) Values below PP = 0.5 or PB = 50. The keys of the taxa are in Table 2

Phylogenetic analyses were performed to estimate the genealogical relationships between these haplogroups using the haplotypes that were detected in this study and the sequences that are designated as subspecies or domesticated in the NCBI GenBank database, which amounts to 64 haplotypes (Table 2). In the ML and BI consensus tree, haplotypes from the wild mexicana population, which was identified as a haplogroup in the network, are basal (C1) (Figs. 2, 3). Although the topology of the consensus tree shows polytomies, we can observe a clade (C2) that integrates intermedia and merriami haplotypes, which were detected as a haplogroup in the network, and a subclade that contains merriami haplotypes, which are linked with the haplogroup with the dominant haplotype Mgmer (Figs. 2, 3). In addition, a large clade (C3) includes haplotypes that were identified in domesticated/commercial, archeological, intermedia, merriami, silvestris, oceola, and mexicana (MGMDgoB/MICH1 and MGMDgoD) individuals, which belong to five haplogroups detected in the haplotype network (Figs. 2, 3). In one polytomy, we found haplotypes of the Mgo, Mgs and MGMDgoB/MICH1 haplogroups. The C3 clade also contains six subclades or expansions: three major ones (SCI, SCII, and SCIII) and three with two haplotypes each (SCIV, SCV, and SCVI). In SCI, the haplotypes corresponding to the haplogroup with the dominant haplotype MICH2 were included (MICH2, MICH5aqui, MICH8coah, Com9, MG80 and MG52) (Figs. 2, 3). SCII, which covers mainly archaeological samples and one merriami individual, corresponds to the haplogroup with the dominant haplotype MgArch. SCIII is comprised of silvestris haplotypes, which correspond to the expansion of the haplogroup with the dominant haplotype Mgs (Figs. 2, 3). Finally, the three subclades with two haplotypes each also correspond to expansions of haplotypes shared by silvestris and osceola turkeys, which in the network are related to the haplotype Mgs through one mutation.

### Analysis of divergence times

Using Bayesian inference, we analyzed divergence times to estimate when the separation of groups and expansions occurred. The results placed the most recent common ancestor (MRCA) of the genera *Meleagris* and *Gallus* at 33.66 million years ago (Mya). *M. gallopavo* and *M. ocellata* shared the MRCA during Pliocene-Miocene time limits [5.35 Mya, HPD (95%) = 2.72–10.17]. Within *M. gallopavo*, the differentiation of the basal group from northwestern Mexico (mexicana population) began during the Pliocene (3.39 Mya) (Fig. 3). The more ancestral haplotypes of the intermedia and merriami turkeys share a MRCA in the Pleistocene (1.65 Mya) (Fig. 3). In clade C2, the subclade, which is composed exclusively of merriami turkeys, originated 1.02 Mya during the Pleistocene and underwent a subsequent diversification with two haplotypes 0.43 Mya during the Pleistocene (Fig. 3). In clade C3, diversification occurred at different times during the Pleistocene. Subclades SCI with haplotypes that were detected in domesticated turkeys originated 0.65 Mya, SCII with merriami and archeological haplotypes diverged 0.39 Mya, and SCIII with silvestris haplotypes originated 0.92 Mya (Fig. 3). The three other subclades diverged 0.08 (SCIV), 0.28 (SCV), and 0.07 (SCVI) Mya (Fig. 3). However, these results should be considered with caution because of the low level of genetic variability in the data analyzed.

### Demographic history

**Fig. 4:**
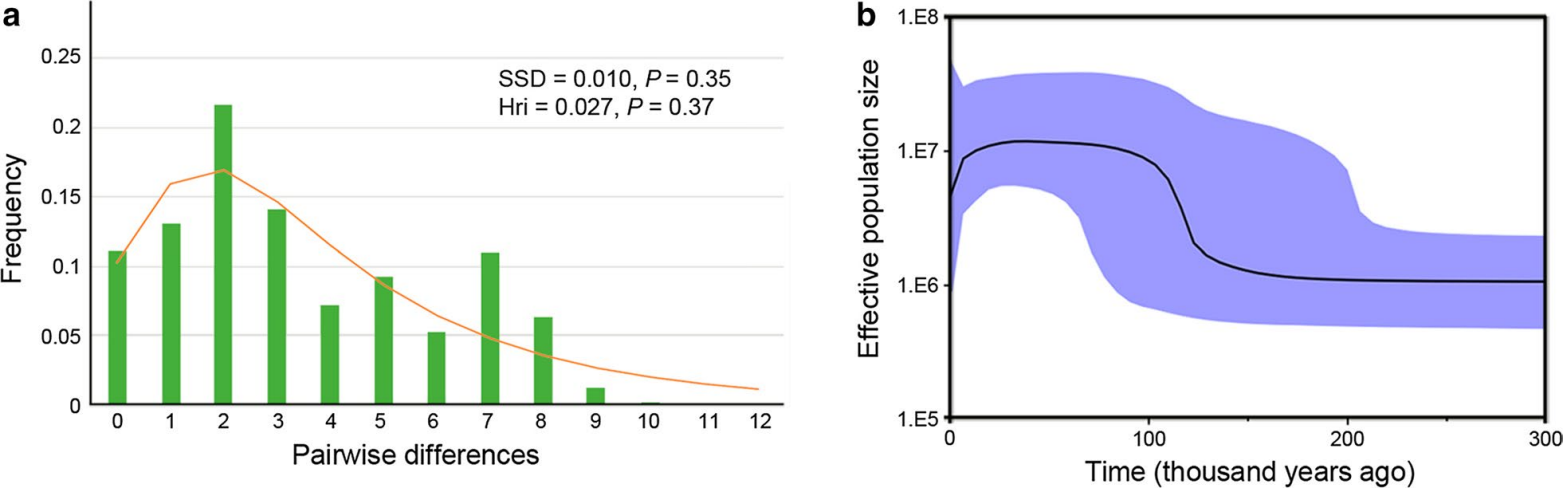
Mismatch distribution and Bayesian skyline plots. Mismatch distribution for the domesticated group (**a**). Histograms correspond to the observed frequencies; the line represents expected frequencies under the sudden expansion model. Bayesian skyline plots shows the population history of *M. gallopavo* (**b**), with the black line indicating median population size estimates expressed in N_*e*_. through time; colored areas represent 95% HPD intervals

Finally, to describe the changes in effective population size through time, we investigated the demographic history of the species. The mismatch distribution graph shows that the highest frequency of pairwise differences is around 2, which indicates that the population analyzed in this study has few mutations, and represents closely-related individuals. The values recovered from SSD (0.010, *P *= 0.35) and Hri (0.027, *P* = 0.37) indicate that the analyzed data fit the sudden expansion model [54] (Fig. 4a).

In the skyline plot, the y-axis shows population size expressed in *N*_*e*_, where *N*_*e*_ is the effective population size and is the generation time, which is approximately one year in *M. gallopavo* [55, 56]. The time axis was scaled using the rate of 0.0046 substitutions/site/million years (SSM) that was obtained in this study. The skyline plot analysis of all *M. gallopavo* individuals as a population shows a demographic reconstruction starting from 300,000 years ago (Kya) (Fig. 4b). Between 300 to 130 Kya, the population remained stable, followed by period of growth and then again a stable period from 90 to 10 Kya. In addition, a slight population decline at approximately 10 Kya was observed in the skyline plot.

## Discussion

### Genetic diversity and differentiation

Based on the analysis of sequences generated for this study and from the NCBI GenBank database, our results show a high level of haplotype and nucleotide diversity which suggests that the turkey population has remained stable with an old evolutionary history [57]. Analysis of metrics of genetic diversity by group revealed particular histories. Domesticated/commercial turkeys showed moderate Hd and low π, which indicates that they originated from a small number of founders [57]. Silvestris, osceola, and intermedia populations showed the highest Hd and a low π, which suggests a bottleneck followed by a rapid expansion [57], whereas the merriami population had a moderate Hd and a high π, which suggests that it has remained stable [57]. Regarding the sequences of mexicana individuals that were obtained from the NCBI GenBank database (n = 27), all except one individual displayed the Mgm haplotype that we detected in this work, which results in this population having both low Hd and π, suggesting a bottleneck (Table 4) [57]. Nevertheless, since we identified five haplotypes for nine mexicana individuals, we believe that increasing sample size and extending the geographic area analyzed would lead to the detection of additional haplotypes.

In agreement with the levels of pairwise genetic differentiation and distribution of genetic variation by AMOVA analysis, approximately three groups of *M. gallopavo* were identified: (1) mexicana, (2) merriami, and (3) intermedia/silvestris/osceola/domesticated/commercial. This result indicates that although these groups share haplotypes, the proportion of unshared haplotypes exclusive to each group is significant and variable [58].

### Haplotype network

**Fig. 5:**
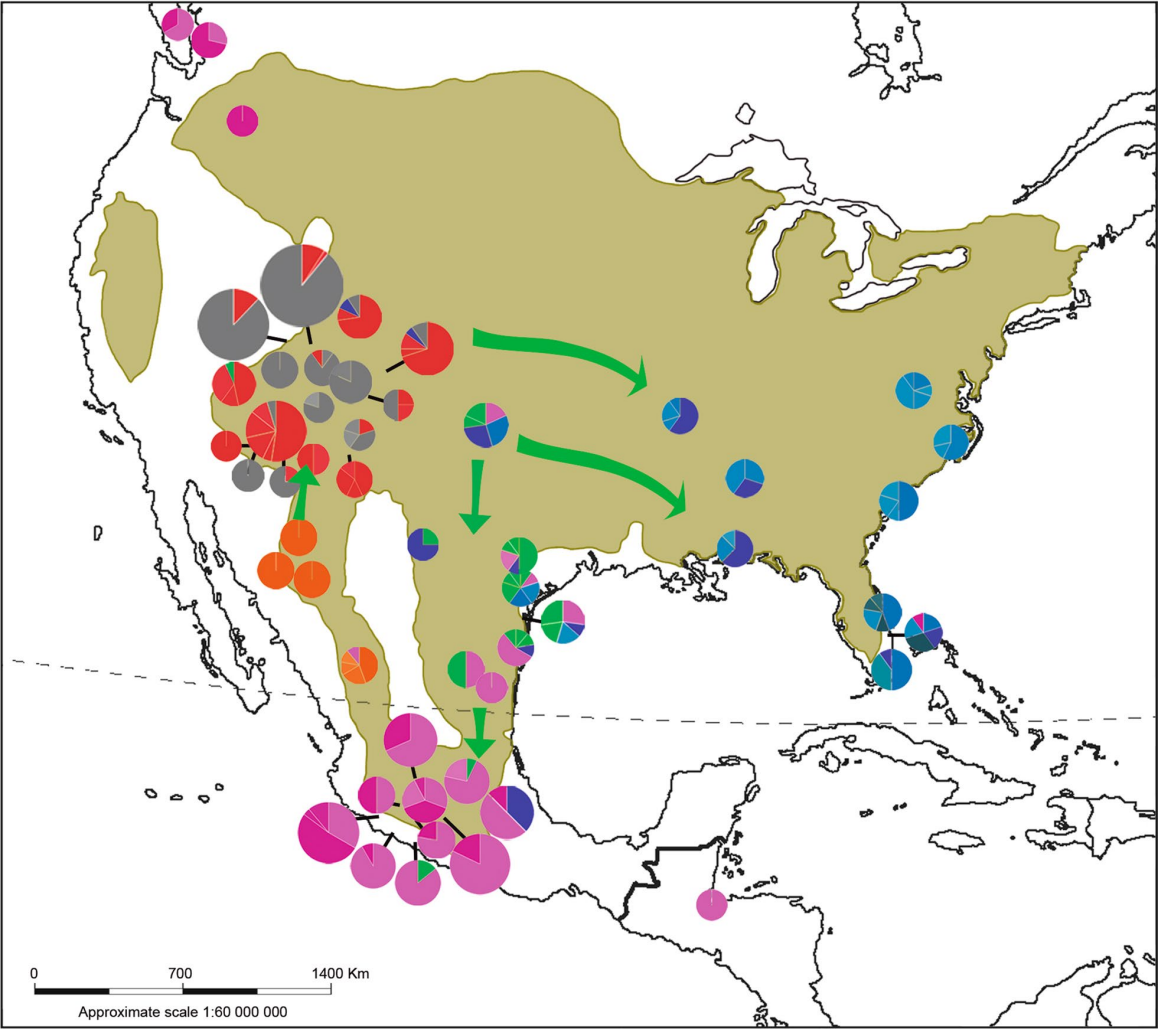
Map of the geographical distribution of *M. gallopavo*. The shaded areas represent the distribution of the species according to Porter and Kirwan [2]. Pie charts represent the geographical distribution of haplotypes found in each sampling locality

We found 64 haplotypes that differ from each other by a small number of mutations, with some of these haplotypes being shared by domesticated and wild populations, which indicates that in *M. gallopavo* there is no sub-speciation; this is in agreement with previous reports for the species [5, 7, 14, 15]. Eight haplogroups, each with a dominant haplotype, were identified. These haplogroups corresponded with the three groups that were identified by the analyses of genetic differentiation and distribution of genetic variation as follows: (1) mexicana (haplogroup with the dominant haplotype Mgm), (2) merriami (haplogroup with the dominant haplotype Mgmer), and (3) intermedia/silvestris/osceola/domesticated/commercial (haplogroups with the dominant haplotypes Mgs, Mgo, Mg, MGMDgoB/MICH1, MICH2, and MgArch) (Fig. 2). Considering that the haplotypes identified in domesticated/commercial turkeys are shared with those in wild turkeys, we designated the third group as mexicana/intermedia/silvestris/osceola. Figure 5 shows the geographic distribution of the haplotypes, which agrees with the three detected groups. It should be noted that the detection of the dominant haplotype Mgs in domesticated turkeys from Mexico and wild populations indicates that its distribution ranges from the central to northeastern and southeastern USA, and from northeastern to central Mexico (Fig. 5). The fact that the dominant haplotype Mgs and its peripheral haplotypes form a star, is typical of population expansions from a small number of founders [57]. This coincides with the genetic diversity analysis of the silvestris population, for which high Hd and π levels and a negative, although not significant, Tajima’s D value (Table 4) were found, which also suggest expansion of the population [57].

On the one hand, our results indicate that the dominant haplotypes MGMDgoB/MICH1 and MICH2 are the main founding maternal lines of domesticated turkeys. On the other hand, since these two maternal lines were detected in individuals of the commercial line Bronze and in domesticated individuals from Canada and the USA, they probably constituted the basis of the current highly selected commercial lines. Our results agree with those of a study that evaluated the genetic diversity of different turkey populations by using single nucleotide polymorphisms (SNPs), i.e. that the heritage turkey varieties Royal Palm and Narragansett and commercial populations derive from the wild mexicana population, that the commercial lines share the same origin, and that possibly specific haplotypes (nuclear DNA) were selected in the modern domesticated turkey [14]. In addition, our finding that some individuals sampled from Guatemala carry the MGMDgoB/MICH1 haplotype indicates that there has been an exchange between northern Mesoamerica and the Maya cultural region, as proposed by Thornton et al. [59]. To confirm this, it would be necessary to analyze individuals from Central America.

### Genealogical relationships and divergence time

Our estimate of the date of divergence between the common ancestor of *Meleagris* and *Gallus* genera i.e. 33.66 Mya agrees with the estimates reported by Claramunt and Cracraft [60]. The separation between *M. gallopavo* and *M. ocellata* 5.35 Mya coincides with the Miocene–Pliocene boundary, which indicates a deep divergence between lineages. We identified different diversification events in turkey particularly during the Pleistocene, which coincides with reports on fossils dated between 0.3 and 2.6 Mya [49]. At the base of the tree, mexicana turkeys (haplogroup Mgm), which currently inhabit northwestern Mexico, are a relict population of *M. gallopavo*. Similar to the results reported by Mock et al. [5] and Speller et al. [7], we found that the mexicana group, represented by the MGM60, MGMDgoA, MGMDgoC, and MGMDgoE haplotypes, is ancestral (C1 in Fig. 3). Our results suggest a diversification process in the mexicana population during the Pliocene (3.39 Mya). We propose that the range of this population expanded towards the north in Arizona and New Mexico and towards the center of the USA. A second group (C2) that comprises the intermedia and merriami haplotypes (haplogroup Mg) originated in the same area (Figs. 2, 3, 5). Currently, merriami and mexicana populations occupy ponderosa pine and pine-oak woodlands of the southwestern USA and northern Mexico, respectively. Apparently, the great deserts of North America, are an efficient geographic barrier for the groups detected in the current analysis. In western North America (southwest of USA and northwest of Mexico), we identified some genetic discontinuities that are associated with the Sonora-Mojave and Chihuahua deserts, which we suppose have isolated and promoted the divergence between mexicana and merriami populations.

Based on our results on the establishment of several haplogroups in the geographical space, genealogical relations and genetic differentiation (Figs. 3, 5 and Table 5), we propose that the mexicana/intermedia/silvestris/osceola genetic group (C3) expanded its range from the center of the USA east to the Atlantic coast and to the south through the Sierra Madre Oriental (SMOr) until it reached the center of Mexico (Figs. 3, 5). Genetic discontinuities have been identified within different vertebrate and plant species in the eastern of USA [61]. Based on our results, in *M. gallopavo* there is no genetic discontinuity in the region (Fig. 5).

However, the occurrence of shared haplotypes in individuals from locations in northeastern Mexico (intermedia) to Michoacan led us to propose that the Trans-Mexican Volcanic Belt (TMVB) may be responsible for the expansion of the range of the C3 group (Figs. 1, 5). We assume that the Mexican Plateau acted as a geographic barrier that limited the contact between populations of mexicana and the C3 group (northeastern-northwestern of Mexico) (Figs. 1, 5).

Finally, we found that the haplotypes present in domesticated turkeys originate from the genetic group C3, which includes the MGMDgoB/MICH1 haplotype and its peripheral haplotypes, and by expansion from the SCI, which includes the MICH2 haplotype and its peripheral haplotypes. Thus, domesticated turkeys do not originate from an extinct subspecies *M. g. gallopavo*.

The presence of the MGMDgoB/MICH1 and MGMDgoD haplotypes in the mexicana relict population, indicate that the mexicana population and the C3 group have been in contact, probably in central-northwestern of Mexico (Figs. 1, 2, 3). However, we did not identify mexicana relict haplotypes in domesticated turkeys, thus this contact between the mexicana population and the C3 group probably took place through the expansion of the species from the center to the northwest of Mexico. It is important to note that a previous analysis based on microsatellite markers showed that the MGMDgoB/MICH1, and MGMDgoD haplotypes are present in the wild mexicana population [11]. In addition, the fact that the samples of wild turkeys collected in 1903 in the Michoacan and Veracruz areas of Mexico shared the haplotypes MGMDgoB/MICH1 and MICH2 indicates that these old and new maternal lines persisted in the wild population of central Mexico until the last century. In contrast to other domesticated species for which events of interspecific hybridization or multiple origins have been observed [19, 26–28, 62, 63], the domestication of the turkey is less complex; according to our results, domestication of this species has a unique origin that is likely in the center of Mexico, whereas Nelson [10] and Leopold [6] both proposed that it was in Michoacan, Mexico. However, the low nucleotide diversity in the D-loop sequence of *M. gallopavo* makes it difficult to determine the center of origin with more precision.

### Demographic history

The rate of substitutions per site obtained in this study coincides with the range of substitutions per site per million years of mitochondrial genes for birds reported in the literature [64]. The multimodal-shaped mismatch distribution suggests demographic stability (Fig. 4a). However, our data also support an expansion (SSD and Hri) that coincides with the population expansion observed in the skyline plot approximately 110 Kya (Fig. 4), which also coincides with the Eemian interglacial period (during the marine isotope stages MIS5e and MIS6d that occurred 133 to 103 Kya [65]), (Fig. 4). Our results of the analysis of genetic diversity (high nucleotide and haplotype diversities) support the observation that the population remained large and stable 90 to 10 Kya (Table 3). The observed population expansion followed by a stable period from 90 to 10 Kya is possibly associated with the expansions detected in the C3 group 80 and 70 Kya (Fig. 3, SCIV and SCVI).

The slight decline of the population about 10 Kya coincides with the cooling during the Younger Dryas. Moreover, it has been reported that 12,900 years ago an extraterrestrial impact occurred in northern North America that contributed to the late Pleistocene megafaunal extinctions and adaptive shifts among Paleoamericans in North America [66]. Environmental changes caused by the combination of these events in North America may have impacted the availability of resources and consequently promoted the declines of the population observed in this study. Although it also is possible that human activities had some impact on the observed demographic decline, there is no evidence of intensive consumption of turkeys by Amerindian tribes (in USA), since they showed preference for large prey during hunting [67–69].

## Conclusions

A large and stable population of *M. gallopavo* occupied a wide geographical distribution from the north to the center of America (USA and Mexico) during the Pleistocene. Due to the expansion of their geographical range and to divergence events during the Pliocene–Pleistocene and Pleistocene–Holocene boundaries, three genetic groups originated within the species: mexicana, merriami and mexicana/intermedia/silvestris/osceola. The MGMDgoB/MICH1 and MICH2 haplotypes and their peripheral haplotypes that belong to the mexicana/intermedia/silvestris/osceola group, are the main maternal lines that were captured for domestication in the center of Mexico (Trans-Mexican Volcanic Belt), the only region of turkey domestication. Domesticated turkeys populations from backyards in Michoacan come from the founding domesticated population. To confirm these results further, sampling of turkeys should be extended to key regions of Mexico (Sonora, Zacatecas, Jalisco, Nayarit, Tamaulipas and the center-south region). Finally, we provide new data on the haplotype diversity that prevails among domesticated turkeys from backyards in Mexican rural communities, with six haplotypes, which, to date, had not been reported in *M. gallopavo*.

## Additional file

**Additional file 1: Table S1.** Haplotypes identified, polymorphic sites and absolute frequencies for each population sampled, including sequences obtained from databases.
